# Supplementary material for: Drip Fertigation Increases Maize Grain Yield by Affecting Phenology, Grain Filling Process, Biomass Accumulation and Translocation: A 4-Year Field Trial
Source: Plants (Basel). 2024 Jul 10;13(14):1903. doi: 10.3390/plants13141903 (PMC11280026; doi:10.3390/plants13141903)
Supplement: Supplementary file 1 [file plants-13-01903-s001.zip › plants-3039451-supplementary.pdf]

Table S1. The basic properties in the 0–30 cm soil layer at the experimental site (measured in June, 2017).

Item	Content	Measurement Methods
Soil pH	8.1	pH meter (1:5 soil/water ratio)
Electrical conductivity (μs cm^{-1})	158.2	Conductivity meter (1:5 soil/water ratio)
Soil NH_4^+-N (mg kg^{-1})	7.2	Continuous AutoAnalyzer3 device
Soil NO_3^--N (mg kg^{-1})	11.9	Continuous AutoAnalyzer3 device
Exchangeable soil potassium (mg kg^{-1})	179.3	Flame photometry method
Available soil phosphorus (mg kg^{-1})	18.7	Molybdenum-blue colorimetry method
Soil organic matter (g kg^{-1})	12.2	Dichromate wet oxidation method

Table S2. The detailed irrigation and nitrogen application times under different treatments in the 2017–2020 growing seasons.

Year	Irrigation Time		Nitrogen Application Time	
	CK and SF	DI and DF	CK and DI	SF and DF
2017	June 27	June 27, July 12, July 19, July 24, August 3, August 16	June 27	June 19, July 19, August 3, August 16
2018	July 21	July 21, July 26, August 2, August 29	June 16	June 20, July 13, July 26, August 13
2019	July 12	July 12, August 14, August 19	June 13	June 16, July 12, July 26, August 19
2020	July 6	July 6, September1	June 22	June 19, July 6, July 27, August 25

Note: Irrigation amounts each time for CK and SF were 90 mm, and for DI and DF were 30 mm. Nitrogen each time for CK and DI was 210 kg ha^{-1}. The nitrogen application for SF and DF was 20%, 30%, 30%, and 20% of the total nitrogen (210 kg ha^{-1}).

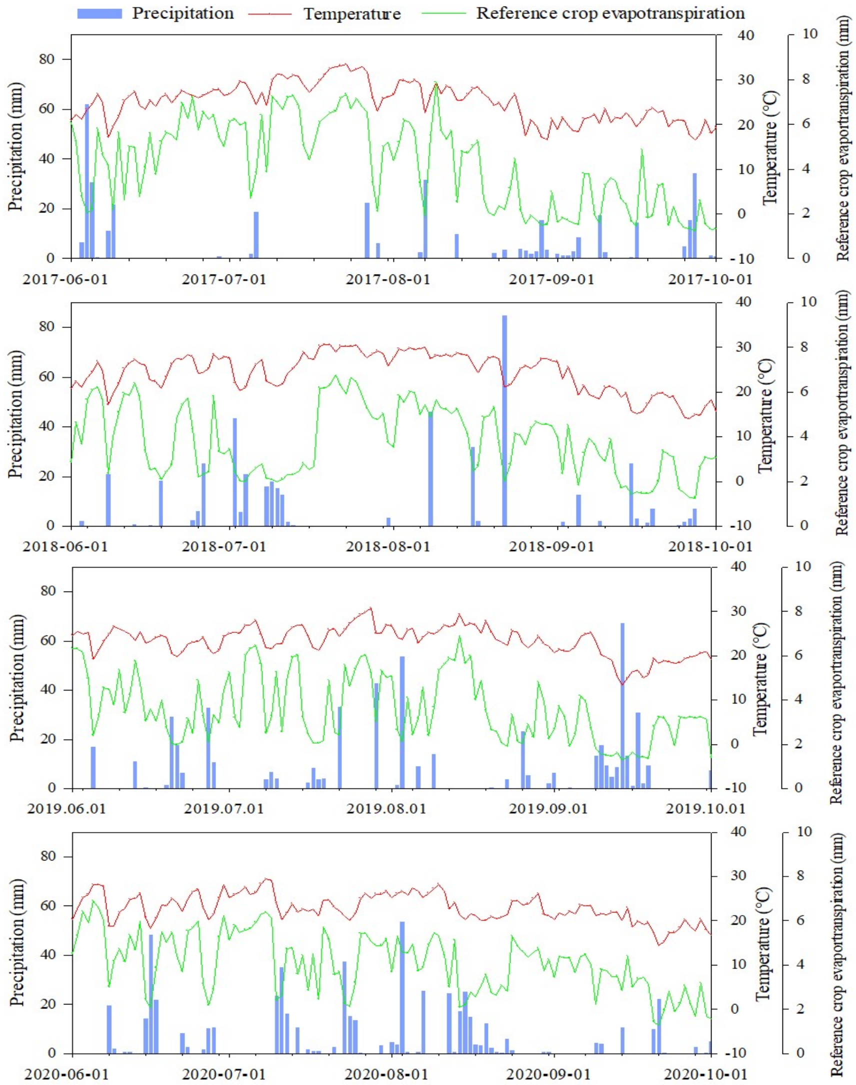

Figure S1. Daily precipitation, average temperature, and reference crop evapotranspiration during the summer maize growing seasons in 2017, 2018, 2019, and 2020.
